# Systematic identification of A-to-I RNA editing in zebrafish development and adult organs

**DOI:** 10.1093/nar/gkab247

**Published:** 2021-04-19

**Authors:** Ilana Buchumenski, Karoline Holler, Lior Appelbaum, Eli Eisenberg, Jan Philipp Junker, Erez Y Levanon

**Affiliations:** The Mina and Everard Goodman Faculty of Life Sciences, Bar-Ilan University, Ramat Gan 5290002, Israel; Berlin Institute for Medical Systems Biology, Max Delbrück Center for Molecular Medicine, Berlin, Germany; The Faculty of Life Sciences and the Multidisciplinary Brain Research Center, Bar-Ilan University, Ramat-Gan 5290002, Israel; Raymond and Beverly Sackler School of Physics and Astronomy and Sagol School of Neuroscience, Tel Aviv University, Tel Aviv 6997801, Israel; Berlin Institute for Medical Systems Biology, Max Delbrück Center for Molecular Medicine, Berlin, Germany; The Mina and Everard Goodman Faculty of Life Sciences, Bar-Ilan University, Ramat Gan 5290002, Israel

## Abstract

A-to-I RNA editing is a common post transcriptional mechanism, mediated by the Adenosine deaminase that acts on RNA (ADAR) enzymes, that increases transcript and protein diversity. The study of RNA editing is limited by the absence of editing maps for most model organisms, hindering the understanding of its impact on various physiological conditions. Here, we mapped the vertebrate developmental landscape of A-to-I RNA editing, and generated the first comprehensive atlas of editing sites in zebrafish. Tens of thousands unique editing events and 149 coding sites were identified with high-accuracy. Some of these edited sites are conserved between zebrafish and humans. Sequence analysis of RNA over seven developmental stages revealed high levels of editing activity in early stages of embryogenesis, when embryos rely on maternal mRNAs and proteins. In contrast to the other organisms studied so far, the highest levels of editing were detected in the zebrafish ovary and testes. This resource can serve as the basis for understanding of the role of editing during zebrafish development and maturity.

## INTRODUCTION

Adenosine to inosine (A-to-I) RNA editing is a post-transcriptional modification that diversifies the RNA sequence from the genomic DNA template. This modification, which is the most frequent form of editing in metazoans (1,2), is catalyzed by double-stranded binding RNA-specific adenosine deaminase acting on RNA (ADAR) enzymes (3–5). The ADAR family of enzymes are evolutionary conserved, with three members represented in mammals. Two of these mammalian ADAR proteins, ADAR1 and ADAR2, are catalytically active, while no activity has yet been detected for ADAR3 so far. It is assumed that ADAR1 is the main enzyme responsible for editing repetitive sites, while ADAR2 mainly edits non-repetitive coding sites (6). Recent evidence indicates that although ADAR1 is critical for normal development in mammals (7,8), the lethal embryonic phenotype can be rescued by deletion of the dsRNA sensor melanoma differentiation-associated protein 5 (MDA5) (9). This observation led to the notion that the main role of ADAR1 is to edit target molecules in order to prevent mistaken identification of endogenous long double-stranded RNAs by dsRNA sensors and consequent triggering of the innate immune response (9–11).

The translational machinery recognizes inosine (I) as guanosine (G). As a result, RNA editing in coding regions of the genome can lead to amino acid substitution and alter protein function. For example, a non-synonymous A-to-I editing event inside the pore of an ion channel can affect neuronal excitability (12). However, this apparently occurs only infrequently, as only a limited number of recoding A-to-I editing sites have been discovered in most species studied so far. RNA editing in non-coding regions is a more common event, with millions of sites identified within paired inverted repeats in human and other species (13–19). The levels of editing of a target may vary between tissues, and can be regulated dynamically as a result of environmental changes such as temperature (20–23), developmental stage (24–26), and in various diseases (27–36).

The rapid development of high-throughput transcriptome sequencing has enabled a global screen of A-to-I editing levels. Considered naively, detection of specific RNA editing events should simply require screening RNA sequencing (RNAseq) data for A-to-G mismatches between the reference genome and the aligned reads. However, the identification of such events is challenging, since it requires the ability to discriminate between RNA editing events, single nucleotide polymorphisms (SNPs), alignment errors, and somatic mutations (37). Therefore, reliable genome-wide atlas of A-to-I editing sites were built for only handful of organisms. Fortunately, sequencing RNA and DNA from the same individual can eliminate most false-positive signals.

Zebrafish (*Danio rerio*) has become a pre-eminent model organism and has been widely used to characterize gene expression and genetic pathways, in order to study developmental processes, organ function and complex behavior in vertebrates. The advantages of this transparent vertebrate model result in the accumulation of genomic data on gene expression, mutations, genetic markers, and information networks (38), among others. However, little is known about RNA editing in zebrafish. Previous genomic analysis revealed that due to genomic duplication the zebrafish genome encodes four ADAR enzymes: ADAR1, ADAR2a, ADAR2b and ADAR3, where ADAR2a and ADAR2b are both orthologs of the human ADAR2 (4,39). Here, we present a rigorous set of A-to-I RNA editing sites in zebrafish obtained by computational tools to identify and categorize editing events with a low false positive rate, including 149 sites in coding sequences, few of which are conserved between humans and zebrafish. The landscape of RNA editing events showed strong association between RNA editing and embryonic development. In addition, we revealed a particularly high level of global editing in zebrafish ovary and testes. These findings suggest a potential role for RNA editing in post transcriptional regulation, cell function and physiology in zebrafish.

## MATERIALS AND METHODS

### Breeding of zebrafish and sample preparation

Fish are kept according to legal regulations of local authorities in Berlin, Germany. For sequencing libraries from embryos, we set up single crosses of wild type individuals (AB strain) and harvested 10 embryos per library after 20 min, 2 h, 4 h, 9 h and 24 h. After 72 h, we removed the head, mainly containing brain tissue, from the body, and stored the samples in Trizol. From the parent fish, we dissected whole brains and muscle tissue from the body and stored them in Trizol. The remaining material of the same adult fish was used to extract DNA.

Different brain regions were obtained by dissecting adult wild type fish (AB strain) on ice. Brain regions were manually dissected from the whole brain in RNAlater using forceps and spring scissors (40), and immediately put into Trizol.

### Preparation of libraries and sequencing

We extracted RNA with Trizol, chloroform and isopropanol according to the manufacturer's protocol, dissolved the pellet in PCR-grade water, checked quantity with qubit and quality with Agilent RNA6000 pico chips on a bioanalyzer 2100 system (Agilent). Libraries for developmental stages and adult whole brain and body were prepared on a robotic system, using Illumina stranded total RNA and mRNA kits. DNA was sheared in TE buffer on a Covaris instrument, then libraries were prepared using the NEBnext Ultra II library prep kit.

RNA from adult brain regions was rRNA depleted using NEBnext rRNA depletion kit for human, mouse and rat. Libraries were prepared with the NEBnext Ultra Directional RNA Library prep kit.

All libraries were sequenced on an Illumina HiSeq4000 machine (150 $ \times$ 2 bp length).

### Sanger sequencing of recoding sites

We designed primers flanking recoding sites on genomic and on mRNA level. We then dissected an adult zebrafish brain and extracted RNA as well as matching DNA from muscular tissue. RNA was reverse transcribed, and target regions of cDNA and genomic DNA were amplified with PCR. Amplicons were sent out for Sanger sequencing, and the results were aligned to RNA reference sequences from ensemble (danRer10) using SnapGene v.5.2.4.

### Quality control and alignment

The quality of the sequence reads was confirmed using the FastQC (41) quality control tool for high throughput sequence data. RNA and DNA reads were separately aligned against the zebrafish reference genome (danRer10) using STAR (42) (version 2.6.0). Reads that were mapped to more than one genomic location were filtered out (outFilterMultimapNmax  =  1). In order to avoid spliced alignment in DNA reads, we used the following petameters: alignIntronMin = 2, scoreDelOpen = −10 000 and scoreInsOpen = −10 000. Unaligned reads were used for detection of hyper-editing events, and aligned reads were used for further analysis. PCR duplicates were marked and removed from the bam files using PICARD (43).

Genomes and gene annotations (Ensembl) were downloaded from the USCS genome browser (44).

### Detection of novel editing sites

In order to reduce possible noise and prevent the detection of single-nucleotide polymorphisms as editing events we utilized RNA and matched DNA sequencing data from the same four individuals. Editing events were detected de-novo using REDIToolDnaRna.py (45). To reduce sequencing errors, 10 bases were trimmed from both ends of the reads, and bases with quality score <30 were discarded. We also demanded coverage of at least 10 reads, and an editing frequency of at least 1%. Sites with a high proportion of multi-mismatches per single position (such as heavily edited A-to-G and A-to-Ts) and sites in homopolymorphic regions (having a high rate of sequencing and alignment errors) were removed.

Notably, whole genome duplication occurred during zebrafish evolution, resulting in ∼30% of the genes represented by two copies (46). To overcome this alignment issue, we filtered out sites with different nearby mismatches. We also focused on sites with at least one additional similar mismatch within a 400bp window (Supplementary Figure S1B). This strategy increases the accuracy of identifying RNA editing sites, but on the other side it may lead us to miss some recoding sites that are present in isolation.

To detect RNA editing sites in coding regions, we annotated all sites, using variant effect predictor (VEP) (47), and then excluded sites in non-coding sequences.

### Hyper-editing analysis

**Figure 1.**
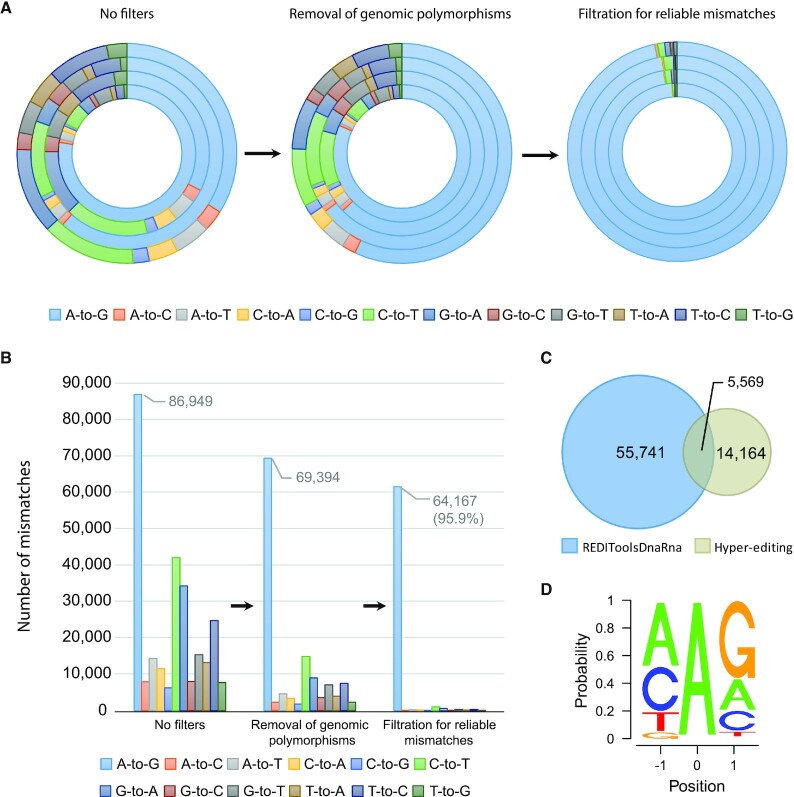
Schematic representation of the process for identifying RNA editing sites in zebrafish. (**A**) The procedure to detect novel A-to-I editing events in zebrafish (identified as A-to-G mismatches between DNA and RNA). Each round of the circle represents a sample, where the total number of any mismatches is represented by the relative proportion. Without applying any filters (left panel) there is a weak enrichment of A-to-G mismatches (32%-81% A-to-G out of all mismatches). Removing potential single nucleotide polymorphisms (SNPs) from the DNA sequencing data (middle panel) reduces the noise level (57–81% A-to-G out of all mismatches). Applying further cutoffs for mismatch enrichment and consistency (right panel) leaves predominantly A-to-G events (91.5–97% A-to-G out of all mismatches). (**B**) Tens of thousands of unique A-to-I editing sites were discovered. In each one of the filtering steps, the total number of mismatches of any type, summed over all four samples, is presented by absolute numbers. (**C**) A-to-I editing sites found in zebrafish by two detection methods: REDITools (45) and Hyper-editing (48). Overall, 76076 unique editing events were identified (overlap of only 5574 sites between the methods). (**D**) A-to-I editing sites exhibited the recognized ADAR motif with guanosine (G) depleted one base upstream and enriched one base downstream, as expected in ADAR targets.

As editing is very abundant in zebrafish, we expected many reads with dense clusters of hyper-editing events to be ignored by standard alignment tools. In order to resolve this issue, we used the method from our recently published study (48), which allows heavily edited reads to be correctly mapped to the reference genome. This led to the identification of 19 733 additional sites, 14 164 of which were detected only by this method (Figure 1C).

### Detection of global editing levels in repeats

The editing index is used to measure the overall editing activity in a sample. Since most editing events take place in genomic repeats, we have modified the human *Alu*-specific editing algorithm (49) to quantify the editing index in different repeats. The editing index is defined as the ratio between the number of A-to-I mismatches and the total number of nucleotides that aligned to the genomic adenosines within the repeats. The higher the index, the more editing occurs in the repeat family element.

Importantly, the editing index is sensitive to read length (49). Longer reads may be mapped, even if they include multiple editing events, and thereby increase the value of the index. Index values should not be compared across studies that used reads of varying lengths.

### Calculating editing levels in coding sites

We applied REDIToolsKnown.py script, part of the REDITools package (45), to quantify the editing levels of detected A-to-I editing events in coding sequence. To reduce sequencing errors, we trimmed ten bases from both ends of the reads. We required that at least 10 reads support the variation and an editing frequency of at least 1%.

### ADAR expression

The expression levels of ADAR transcripts were quantified using Salmon (50). Ensembl genome browser (version 91; EMBL-EML) was used for gene annotation. The tximport (51) package (version 1.0.3) in R was used to transfer Salmon output from transcript level to gene level abundance.

### Data analysis

The statistical analysis was done using R (http://www.r-project.org/). The Wilcoxon rank test was used to test statistically significant differences (95% confidence level) between two groups.

### Data sets

Ovary and testes RNAseq dataset (GSE96603) from Armant *et al.* (52) (no editing differences were observed between the uranium exposed and control groups, data not shown). Heart RNAseq dataset (SRP173044) from Klett *et al.* (53) (no editing differences were observed between different post cryoinjury samples, data not shown). Liver, skin and brain RNA seq dataset (SRP065208) from Irizar *et al.* (54) (no editing differences were observed between 6, 12, 24, 36 and 42 months, data not shown). To make all datasets comparable, we considered paired-end samples as two separate single-end samples.

## RESULTS

### A clear A-to-I RNA editing signal in zebrafish

To detect RNA editing events in zebrafish, we sequenced RNA and the accompanying DNA from four individual zebrafish brains, where editing is common in other animals studied so far (55). The DNA-seq data ($ \approx 7.4\; \times \;{10^8}$ reads) and the RNA-seq data ($ \approx$1.8 $ \times {10^8}$ reads) were aligned against the zebrafish reference genome (danRer10), and screened for possible editing sites by REDITools (45) (see methods). The specificity of the detection scheme is evaluated by comparing the A-to-G mismatches, which could result from A-to-I editing, with other mismatches. Without applying any filters, 271 986 mismatch sites (supported by DNA reads) were detected, of which 86 949 ($ \approx 32\% )\;$were A-to-G (Figures 1A and B). The next most common type of mismatch was C-to-T (15.5%, Supplementary table S1). The high rate of false-detection may result from random sequencing and alignment errors, or genomic polymorphisms, among other sources for such mismatches (37). To remove strain specific polymorphisms and somatic mutations, we aligned matched DNA sequencing data to the reference genome excluding all non-homozygous genomic sites found in at least one of the four samples. This step reduced the total number of detected mismatch sites to 129 296 sites, of which 53.7% (69 394) were A-to-G sites (Figures 1A and B). We then kept only reliable sites, meeting defined cutoff criteria for mismatch enrichment and consistency (see methods). First, we excluded clusters of sites that include multiple substitution types. Such non-homogeneous clusters are a typical signature of alignment errors. Next, we discarded isolated mismatch sites having no additional, same-type, mismatch within 400 bp, as it is well known (in other organisms) that RNA editing events are often accompanied by neighboring editing sites (48). These steps resulted in a total of 63 837 mismatches (Figures 1A and B), most of which (96%, 61 310 sites) were A-to-G, with a rather low noise level of 1.5% (estimated by the number of C-to-T mismatches). The noise level may actually be an overestimate, since some of the C-to-T events may be the result of RNA editing by APOBEC1 proteins (56,57). The detection procedure is illustrated in Supplementary Figure S1A.

ADAR enzymes are known to edit dense clusters of several adenosines, resulting in extensively edited RNA molecules. Standard editing detection methods often fail to identify such events, since these reads are frequently discarded by typical alignment tools, but they may be identified using a previously published method (48). Applying the hyper-editing pipeline to $ \approx$1.8 $ \times {10^6}$ unmapped reads ($ \approx$9.7% of all reads, after STAR (42) aligner) in the RNA-seq dataset revealed 19,733 unique A-to-G sites (total 77 686 editing events) with a very high specificity. The next most abundant mismatch was T-to-C (<0.1% of the detected sites, Supplementary Figure S1C). This method allowed us to identify 14 164 additional sites (Figure 1C).

Together, these analyses have yielded a total of 75 474 putative A-to-I editing events. These editing sites exhibited the known ADAR deamination motif, with a strong depletion of guanosine (G) immediately upstream of the edited site, and some enrichment of G immediately downstream (Figure 1D). Next, we checked whether there is any difference in the editing motif in repetitive and non-repetitive elements and found them to be identical.

### A-to-I RNA editing is rare in coding sequences

**Figure 2.**
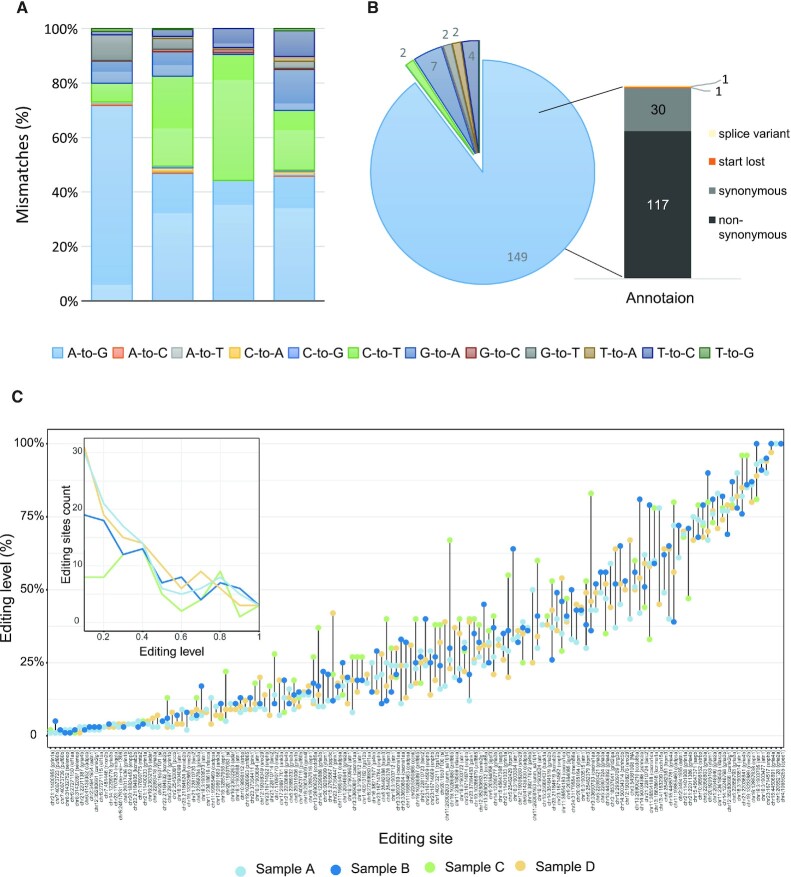
A-to-I editing sites within coding sequences. (**A**) Previously described pipeline after applying our filtering scheme for coding regions (Ensembl (88) annotations). The analyses were performed on the four brain samples. Low specificity of A-to-G mismatches was observed, suggesting a high false-positive rate. (**B**) Relative proportions of the total number of mismatches detected after considering only recoding sites appearing at least in two out of four samples. Most of the detected mismatches were of the A-to-G type. (**C**) Editing levels in 149 A-to-I editing events in CDS, colored by the sample in which it was detected.

Although most RNA editing events take place in the non-coding regions of the genome, editing events that do occur in coding sites can alter the protein sequence, and thus expand the proteome diversity. Out of our 75 474 A-to-G sites, 45.4% (34 254 sites) were located in gene regions with 23 774 (69.4%) in exons, and only 608 (2.6%) in coding regions. Out of these 608 A-to-I editing sites, 88 (14.5%) were detected by the hyper-editing algorithm, with 521 sites detected by the approach described here. The false-positive rate in the coding region, estimated by the number of C-to-T mismatches (the dominant mismatch in all four samples), is rather high, at about 7–47% (per sample, Figure 2A). In order to improve accuracy, we considered only coding sites that appeared in at least two out of the four samples. This step yielded a total of 149 (88.7%) putative A-to-I editing sites (Figures 2B, C, Supplementary Figure S2A and Supplementary table S2), with a residual noise of only 4% (seven mismatches of G-to-A, Figure 2B). Notably, most of these sites exhibit a consistent editing level across samples (median coefficient of variance (CoV) = 0.24). Previous study have identified only 18 of these sites (39) (Supplementary table S3). Interestingly, eleven of the 149 A-to-G editing sites are evolutionarily conserved between humans and zebrafish (Supplementary Figure S2B and C), suggesting that this editing confers a selective advantage. The conserved sites lie within five neuronal genes: *gria2* (*glur2*), *gria3 (glur3)*, *gria4* (*glur4*), *cadps* and *cacna1d* (Supplementary Table S4). In mammals, the role of editing in the genes encoded for glutamate ionotropic receptor AMPA type subunit 2, 3 and 4 is controlling the permeability of the channels, the role of editing in *cadps* gene is to promote dense core vesicle exocytosis (58), while the role of the RNA editing in *cacna1d* is to modulate SCN rhythmicity (59).

The 149 identified A-to-G editing sites lie within 55 genes. Notably, 51 genes are edited both in zebrafish and human, but not in the same positions (RADAR database (14)). Five of them are conserved in mammals (60) and therefore may have maintained a sequence conservation during evolution. Evaluation of gene ontology by the PANTHER (61) classification system, revealed a significant enrichment of genes for ion transport (FDR < 9e–4). These include various glutamate receptor genes (gria2a, gria2b, gria3a, gria3b, gria4a, gria4b, grin1a and grin1b), a potassium channel (kcnab2b), and calcium channels (cacna1da, cacna1aa, kcnma1a and trpm3). This goes in line with previous studies, showing that RNA editing has an important role in signal transduction and neuronal function (12).

Out of the 149 A-to-G coding sites, 117 were identified as non-synonymous sites that lead to amino-acid substitution (recoding sites, Figure 2B). The index, calculated as total number of guanosines divided by the total number of guanosines and adenosines, of these non-synonymous sites was 42.9%, which is rather high. The sites were well covered, with a median coverage of 60 reads. Interestingly, one of the 149 detected A-to-G sites was start-loss (variant effect predictor, VEP (47)). Notably, ADAR1 mRNA is auto-edited and contains two recoding non-synonymous events (chr16:23632195, chr16:23632263). The editing level of these two sites is rather low, median of 5% and 10% respectively, with a median coverage of 72 and 82 reads, respectively. Auto-editing of ADAR2 in mammals and dADAR in Drosophila is a well-studied auto-regulatory mechanism (62,63). Interestingly, we identified a nonsynonymous editing site in ADAR2b mRNA. However, since we detected this site in only one of the samples, it was filtered out by our analysis. It is therefore possible that an auto-regulatory mechanism exists also in zebrafish ADAR2b, but unfortunately, we do not have enough information to corroborate this hypothesis.

Next, we sequenced RNA from six different brain regions (olfactory bulb, telecephalon, optic tectum, diencephalon, cerebellum and hindbrain), and revealed that some of the editing sites show variability in editing levels in different brain regions (Supplementary Figure S3A). Interestingly, the non-synonymous *gria2a* editing site, which replaces the genomically encoded glutamine (Q) with arginine (R) and is known to be essential to normal brain function (64) is also highly edited in all brain regions (chr1: 20652128).

### Genomic repeats are highly edited in zebrafish

**Figure 3.**
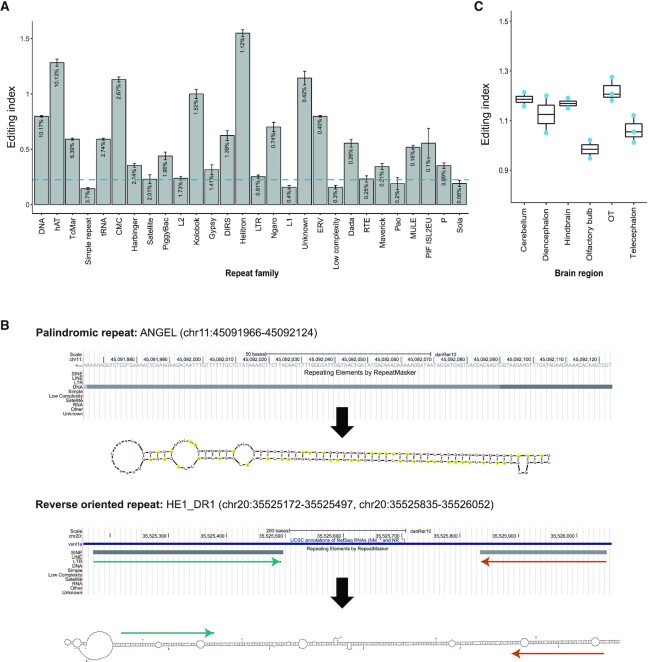
Editing in zebrafish repeats. (**A**) RNA editing index (defined as the editing level averaged over all edited and unedited adenosines) measured across all repeat families. The editing index varies across repeat families and is not enriched in a specific repeat family. The repeats ordered by their abundance in the genome (abundance percentage is shown within each bar). The blue line represents the maximal index of the next most frequent substitution (C-to-T mismatch in low complexity repeat family, value of 0.32%). (**B**) Prediction of the secondary structure of the most edited repeats. Upper structure represents the palindromic repeat, ANGEL, a member of hAT repeat family (using mfold (89)). Here, we show the most prominent single ANGEL repeat, with 62 unique editing sites (62/63 adenosines). Edited adenosines are marked in yellow; The lower structure represents a prediction of the secondary structure (using mfold (89)) of two reverse oriented HE1_DR1 repeats, members of SINE elements (tRNA-V family). (**C**) Global repeats editing index measured across six different brain regions.

In most species, the vast majority of editing events takes place in repetitive elements, which are likely to form long double-strand RNA (dsRNA) structures, required by an ADAR enzyme (19). In humans, for example, editing is highly abundant in *Alu* elements, which are a primate-specific repeat (15–18). These short elements (about 300 bp long) are widely spread across the genome, frequently with a reverse oriented *Alu* repeat nearby, so that together they can form strong dsRNA structures. In mouse, the vast majority of editing sites reside in B1 and B2 repetitive elements (65), which also have a tendency to form dsRNA structure. To assess the overall RNA editing levels in zebrafish, we calculated a global editing index (49) (the number of 'G’s aligned to adenosines, weighted by the expression level of all adenosine positions) for each specific repeat family. A higher index indicates a higher level of overall editing within the repeat family elements. Interestingly, unlike the situation in primates and mice, editing in zebrafish is not enriched in specific repeat families, but appears to be spread across the majority of repeats (Figure 3A). This type of scenario with extensive editing in various repeats has been observed previously in *Cephalopods* (66).

The abundant editing seen in zebrafish can be explained by the presence of many palindromic sequences in repeat families. These palindromic repeats can fold over on themselves to form stable double strand RNA structures (Figure 3B), even in the absence of a neighboring repeat. Other repeats, such as HE1_DR1, a member of the tRNA family, SINE class of repeats, are edited as the result of dsRNA formation with a nearby, similar, reverse oriented repeat.

Accordingly, we grouped all repeat families for which the A-to-G signal was 2-fold higher than the next most frequent substitution (to ensure we include in the index mostly bona fide editing and not mismatches of other technical or biological source, Figure 3A) and calculated the global repeat index over all of these regions ($ \approx$ 4.5 $ \times {10^8}$ bp). The average editing index is calculated to be 0.93% in adult zebrafish brain, averaged for four brain tissues, (i.e. one in a hundred adenosines is deaminated into an inosine). Our brain region sequencing revealed that most of the regions exhibit a similar global repeat editing index (Figure 3C), except for the olfactory bulb where editing is relatively low. This is not entirely surprising, since the levels of ADAR3 (who is considered to be catalytically inactive and competing with editing activity (6,67)) expression are significantly higher in this part of the brain (Supplementary Figure S3B).

### RNA editing landscape across zebrafish development

**Figure 4.**
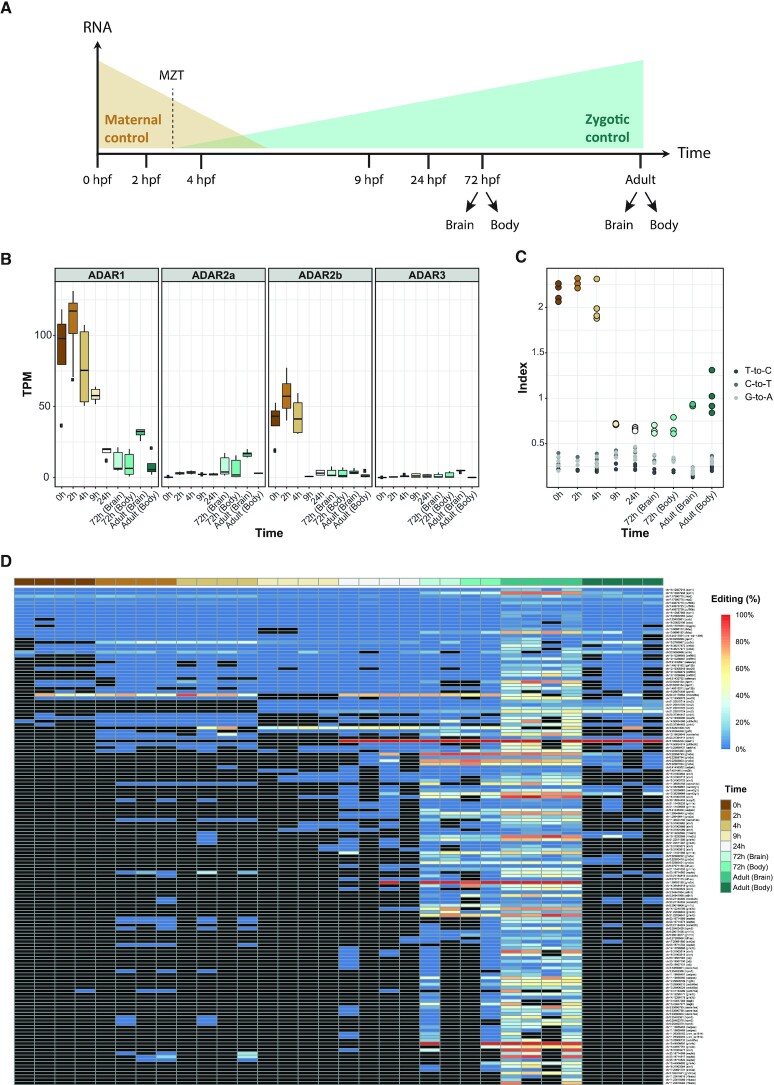
RNA editing during zebrafish development. (**A**) Graphical representation of zebrafish developmental stages. The sequenced samples are presented across a developmental time course. The early stages of the zebrafish development are driven by maternally supplied mRNA, followed by a slow activation of the zygotic genome, with a short period of maternal to zygotic transition (MZT). Brain adult samples are the same we used to detect the recoding sites. (**B**) ADAR expression levels suggest that ADAR1 and ADAR2b are overexpressed in the initial steps of embryonic development. (**C**) The repeats editing index was used to assess the editing activity across developmental stages. The editing index shows a similar pattern, with high levels of editing in early developmental stages, suggesting that ADAR1 and ADAR2b is are responsible for the alteration in editing levels. (**D**) Heat map of RNA editing frequency of 149 A-to-I sites obtained from the recoding detection. Only sites covered with more than 10 reads in at least one sample are shown. The color of each rectangle represents the editing level (white denotes 0% editing; blue denotes 100% editing). Black rectangles denote editing sites supported by <10 reads or those that had no coverage. The highest editing levels were found in samples from 72 h embryos and adult brain. Most of the sites in the early developmental stages had no coverage at all.

The developmental processes underlying the transformation from fertilized egg to adult fish have been studied extensively, and multiple gene networks that drive crucial steps of the zebrafish development have been identified (68–72). However, A-to-I editing in this model has scarcely been studied (39), and editing changes along development have not been thoroughly characterized in vertebrates. The first steps in embryonic life are controlled by maternal mRNAs and proteins that are deposited in the egg during oogenesis (73,74). Following this stage, ∼3.3 h post fertilization (hpf), the period of maternal-to-zygotic (MZT) transition commences, in which maternal transcripts begin to be eliminated, and *de novo* transcription is initiated (74). Hence, transcriptional control of gene expression by the embryo only becomes possible after MZT, a stage at which the embryo already has >1000 cells, and consequently post-transcriptional mechanisms of gene regulation are particularly important during early zebrafish development. To study the editing patterns during embryogenesis, we sequenced six different stages of early zebrafish development: one-cell stage (zygote, 0 hpf), 64-cell stage (cleavage period, 2 hpf), sphere stage (blastula period, 4 hpf), 90%-epiboly stage (gastrula period, 9 hpf), prim-5 stage (pharyngula period, 24 hpf), and the body and brain from the protruding-mouth stage (larval period, 72 hpf). All offspring was produced by the same adult fish to reduce SNP heterogeneity. In addition to these samples, we also analyzed $ \approx$1.6 $ \times {10^9}$ reads (150 $ \times$ 2 bp length) from adult brain and body RNA sequencing data (Figure 4A).

As the first stage of characterizing the editing process, we examined the expression of the four encoded ADAR genes in zebrafish. Notably, in metazoans, the ADAR enzymes are highly conserved, with a difference in the expressed ADAR isoforms between species (75). We observed extremely high levels of ADAR1 and ADAR2b expression in the first stages of the embryotic life span, when the embryo relies upon maternal mRNAs and proteins. These levels decreased in the later phases when the zebrafish produces its own proteins (Figure 4B). In contrast, ADAR2a levels increased only 72 hpf, and were found mainly later, in the adult brain (Figure 4B).

To compare global editing activity across zebrafish development, we calculated the repeats editing index at the different stages. As expected, the total editing index mirrored the trend of ADAR1 expression at all embryotic time points (Figure 4C). This is consistent with the notion that in zebrafish, like in mammals, ADAR1 is the main enzyme responsible for editing of repeats (6). It was interesting to note that the global editing index is particularly high in the initial stages of embryonic development (0, 2 and 4 hpf), and then suddenly decreases (Figure 4C). Therefore, it is possible that this editing activity is inherited from the adult's gametes.

In addition to the developmental samples sequenced, we also analyzed publicly available data from 18 time points along the embryonic development in zebrafish (76), ranging from the one-cell stage to 5 days post-fertilization. Four time points are before zygotic transcription starts (0, 0.75, 2.25 and 3 hpf), four during gastrulation (4.3 hpf – during MZE transition, 5.25, 6 and 8 hpf), and three time points from each of the two following periods: somitogenesis and pharyngula. In addition, four samples were collected every 24 h from 2 days post-fertilization (dpf), to 5 dpf. Again, the expression of ADAR proteins mirrored the trend in global editing index (Supplementary Figures S4A and B). Interestingly, as more time points were investigated, a gradual decrease in the repeats index could be detected during the gastrulation period. Importantly, the index is ∼1.5 higher than was observed in our dataset, probably due to the different sequencing protocol (total RNA-seq versus mRNA-seq).

Although most A-to-I RNA editing events take place in genomic repeats, the rare sites within coding regions are of particular significance, as they can cause a change in the protein function. In order to assess the frequency of these events, we used REDITools to examine the editing levels of the sites obtained from the recoding detection (see above) (45). Out of the 149 previously detected sites, 74 were covered by at least 10 reads (Figure 4D), 38 (51%) of them were non-synonymous. The vast majority of the sites were found in genes that had no coverage in the early steps of the zebrafish development (88% in 0 hpf, 78% in 2 hpf). Notably, this approach is somewhat biased since we used only brain samples to detected de-novo editing sites, as we don’t have matched DNA-seq data from other embryonic stages. Interestingly, ADAR2a (ortholog of the mammalian ADAR2), which is considered to be responsible for most A-to-I editing in recoding sites, was more highly expressed in 72 hpf embryo samples and in adult brain (Figure 4B). This supports the observation that more recoding events, with higher levels of editing, were detected at these time points.

### Elevated RNA editing in zebrafish ovary and testis

**Figure 5.**
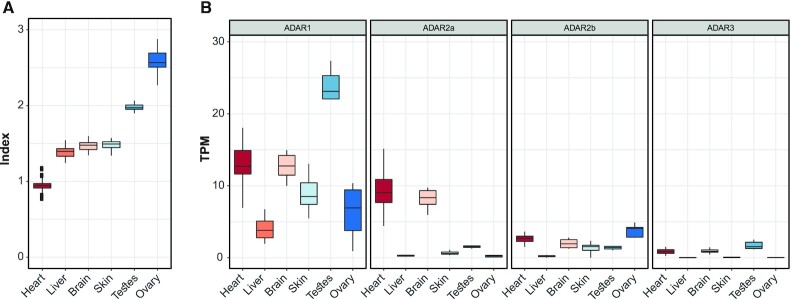
RNA editing across zebrafish tissues. (**A**) Global repeats editing index, the weighted editing level over all adenosines in the most edited repeats, as measured in six different tissues. The editing levels vary between the different tissues and were significantly elevated in testes and ovary. (**B**) Expression levels of ADAR enzymes in each tissue.

The RNA editosome has been extensively studied in a variety of human and vertebrate tissues (26,77,78). Here, we systematically screened for A-to-I RNA editing alterations in 185 samples taken from six different adult zebrafish tissues taken from the GEO database, namely brain (24 samples), heart (90 samples), liver (24 samples), ovary (10 samples), skin (25 samples) and testes (12 samples). In total, we analyzed $ \approx \;8.97\; \times \;{10^9}$ RNAseq reads (read lengths 50–75 bp). In order to compare A-to-I RNA editing levels between these tissues, and minimize the technical variability, we trimmed all reads to 50 bp length. The results indicated that global editing index levels vary across tissues (Figure 5A) and surprisingly, are highest for ovary (average of ∼2.4%) and testes (average of ∼1.99%). This relative ranking in the zebrafish differs from the situation in humans, where most editing takes place in brain sub-tissue and arteries (49). All tissues exhibited the recognized ADAR motif (Supplementary Figure S5). Moreover, while ADAR1 is probably the main contributor to global editing, we observed a reduction of ADAR1 levels and high expression of ADAR2b in ovary (Figure 5B). This suggests that ADAR2b may contribute to the elevated global editing index seen in the zebrafish ovary.

Clustering analysis of the editing levels of the 149 detected recoding editing events across tissue types, revealed a clear separation between brain and non-brain tissues (Supplementary Figure S6A), is in agreement with previously reported human data (78). Interestingly, a clustering analysis of all 757 717 putative A-to-I editing sites, revealed an almost perfect separation between tissues (Supplementary Figure S6B).

## DISCUSSION

This study provides a first description of the comprehensive set of editing sites in zebrafish, comprising tens of thousands of unique A-to-I editing events identified in brain RNAseq samples. Our computational approach provides a clean signal, with a minimal rate of false-positives (estimated by the number of A-to-G conversions compared to other mismatches). Most of the editing events are found in transcribed genomic repeats, as long double-strand RNAs, which are the major substrates for ADAR1. Accumulation of these dsRNA structures was recently shown to trigger the innate immune response, with the conclusion that the main role of ADAR1 is to edit these structures and hence block the immune response (9–11). Our results confirm an abundance of potentially double stranded structures, formed since many of the repetitive elements identified in zebrafish are approximate palindromic sequences, which may fold and create a tight dsRNA structure. A connection between opening these structures and the immune response has not yet been studied in zebrafish. More analyses are needed to discern whether the non-specific active of zebrafish ADAR1 is for reducing the immune response or is a defense system against foreign dsRNA.

Although editing is very common across the genome, RNA editing is rare in coding sequences in zebrafish. This is probably due to the presence of an active mechanism for eliminating evolved editing sites from coding regions if they are not beneficial to the organism. Of the 149 A-to-I editing sites identified in coding regions, 117 are non-synonymous, meaning that they may modify the function of the coded protein. Previous studies have shown that editing is critical to the function of nervous system, and essential for neurotransmission (79–82). In support of this observation, our editing set is significantly enriched with ion transporters, where even small sequence changes can dramatically influence the permeability and plasticity of an ion channel (12). Notably, some of the sites identified are conserved between zebrafish and humans, which clearly demonstrates the association with an evolutionary benefit.

Early embryonic development, before zygotic genome activation, is directed by maternally deposited RNA (73,83,84). Although there have been many studies of the role of maternal mRNAs and proteins deposited in the egg during oogenesis in different model organisms (85,86), certain genomic phenomena, including the RNA editing profile during development, have been poorly characterized. Here, we show a clear change in the editing levels and the levels of *ADAR1* and *ADAR2b* mRNA expression during zebrafish development (Figure 4). Extremely high levels of editing were detected in the initial stages of the embryotic life span, when the embryo relies on maternal mRNA and proteins. This could be attributed to activity of the ADAR2b transcript (ortholog of mammalian ADAR2). We propose, based on this observation, that one of the possible purposes for the robust ADAR activity is to transfer specific information via maternal RNA to the embryo, or to provide an additional layer of posttranscriptional regulation before activation of the zygotic genome. Another potential possibility is that RNA editing affects the stability or translation of edited maternal mRNAs (indication for that is given at Supplementary Figure S7). Interestingly, our editing analyses in zebrafish revealed unusually high editing in the ovary and testes (Figure 5). Thus, another possibility is that RNA editing may be used to introduce variability among zebrafish offspring, without relying on somatic changes in the parental genome, as already been shown in corals (87). This could not be fully explained by expression of ADAR enzymes and further analysis will be needed in order to understand the implications of this phenomenon. These results may provide the basis for the identification of new regulatory mechanisms in zebrafish development.

## DATA AVAILABILITY

All raw sequencing data created for this study is available for download from NCBI short read archive (SRA) database under the accession number PRJNA674002. Other sequencing data used in this study are publicly available.

## Supplementary Material

gkab247_Supplemental_Files
