# Supplementary material for: Systematic identification of A-to-I RNA editing in zebrafish development and adult organs
Source: Nucleic Acids Res. 2021 Apr 19;49(8):4325–37. doi: 10.1093/nar/gkab247 (PMC8096273; doi:10.1093/nar/gkab247)
Supplement: gkab247_Supplemental_Files [file gkab247_supplemental_files.zip › SUPPLEMENTARY FIGURES.pdf]

SUPPLEMENTARY FIGURES

Supplementary figure 1

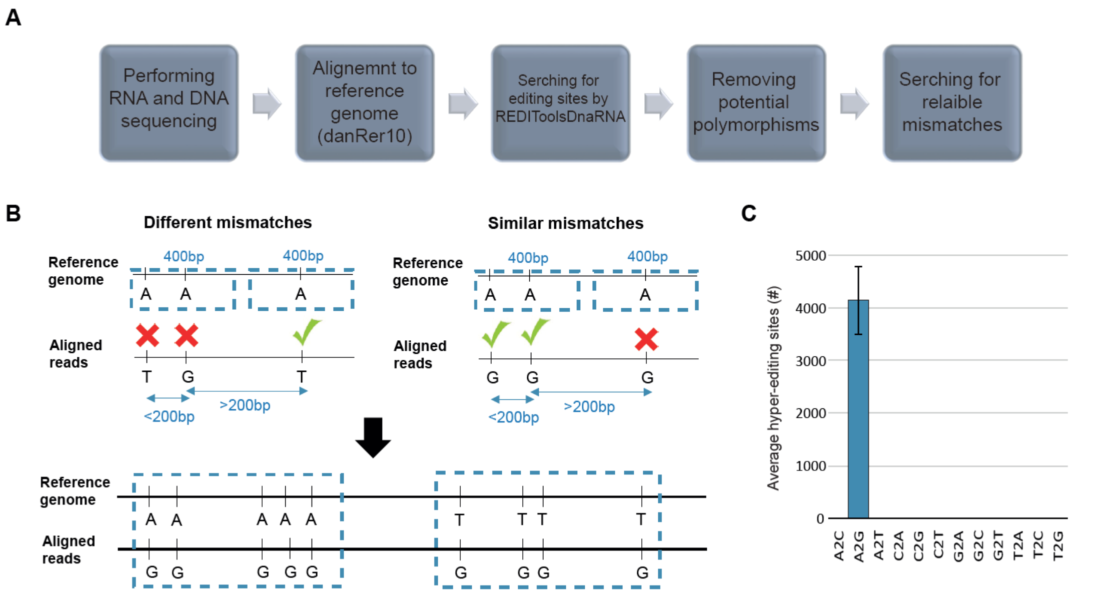

Supp. Figure 1: Overview of editing detection pipeline. (A) The general pipeline for identifying novel editing sites in zebrafish brain, using matched DNA and RNA sequencing data. **(B)** Schematic representation for choosing reliable editing sites which meet cutoffs for editing enrichment and consistency. In the first step we removed clusters of editing sites with potential alignment errors, particularly we excluded clusters of sites that includes multiple substitutions types within 400 bp. We then searched for mismatch accompanied by neighboring same mismatch within the same window of 400 bp. **(C)** Count of hyper-editing events in zebrafish brain samples. Most of the detected mismatches were of A-to-G type.

Supplementary figure 2

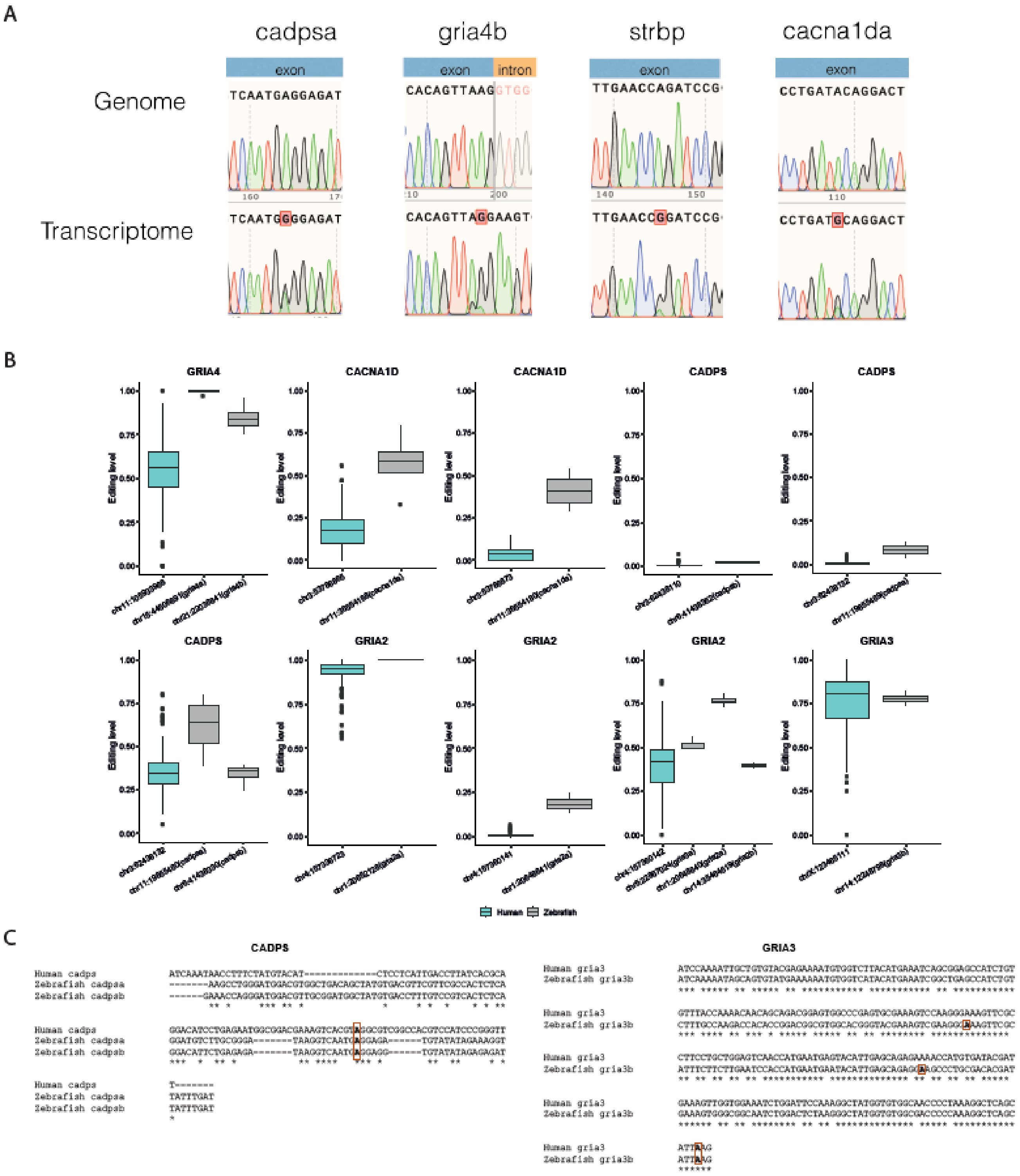

Supp. Figure 2: A-to-I editing sites within coding sequences (A) Validation of cadpsa (chr1:19855459), gria4b (chr21:22036840), strbp (chr10:9898151) and cacna1da (chr11:36554179) editing using direct Sanger sequencing (see methods). **(B)** Conserved editing sites between zebrafish and humans. Compared cohorts of zebrafish editing levels (grey) and human editing levels

(light blue) of conserved editing sites. Human editing levels were calculated on brain samples from GTEx donors (205 samples). **(C)** Multiple alignment (using Clustal Omega) between human and zebrafish cadps genes (left) and gria3 genes (right). The editing events are marked in orange triangles).

Supplementary figure 3

A

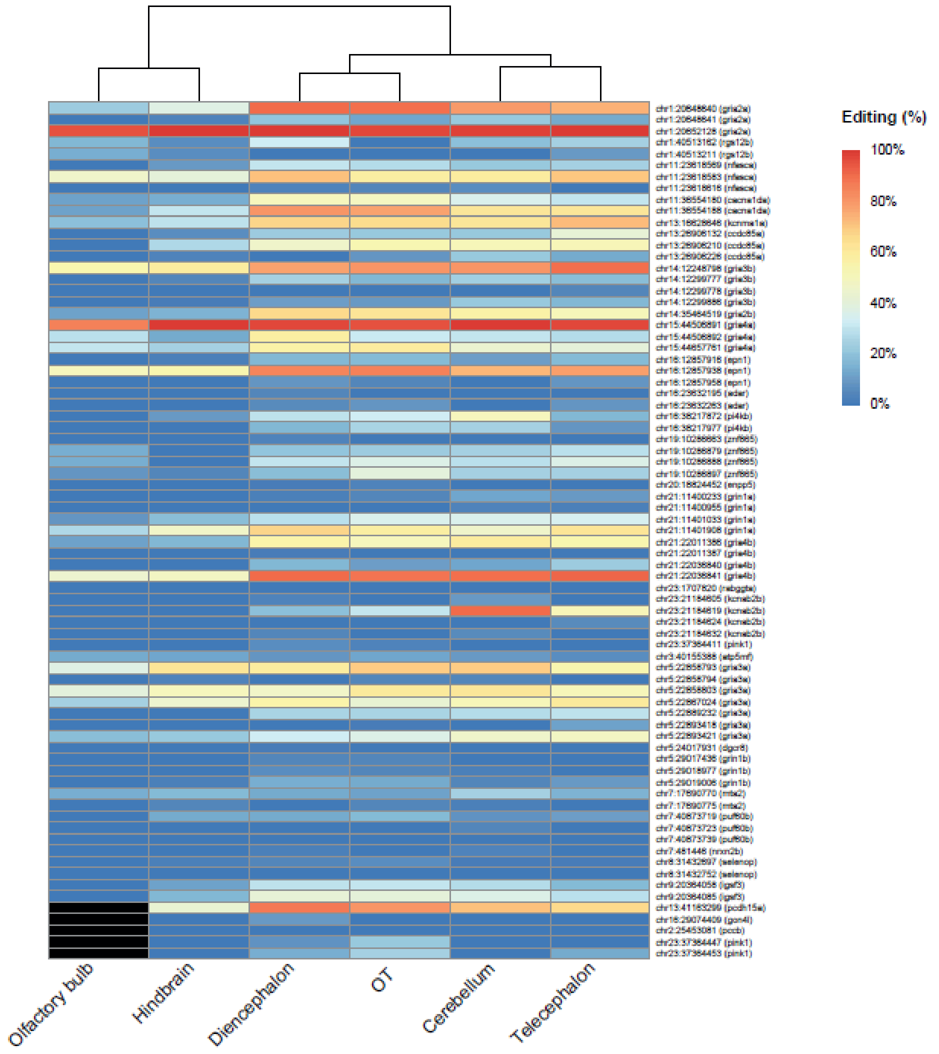

B

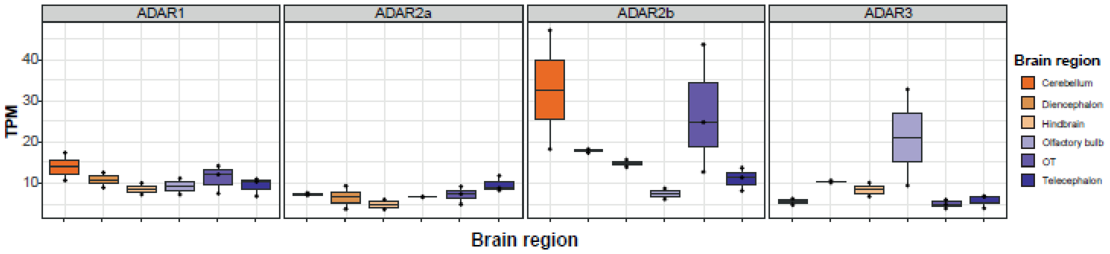

Supp. Figure 3: RNA editing in different brain regions. (A) Heat map of RNA-editing levels of the 149 detected coding editing events (Supplementary table 5). Only sites covered with more than 10 reads are shown. The color of each rectangle represents the editing level (white denotes 0% editing; blue denotes 100% editing). Black rectangles denote editing sites supported by less than 10 reads or those that had no coverage. **(B)** ADAR expression levels distribution of ADAR1, ADAR2a, ADAR2b and ADAR3 for six brain regions.

Supplementary figure 4

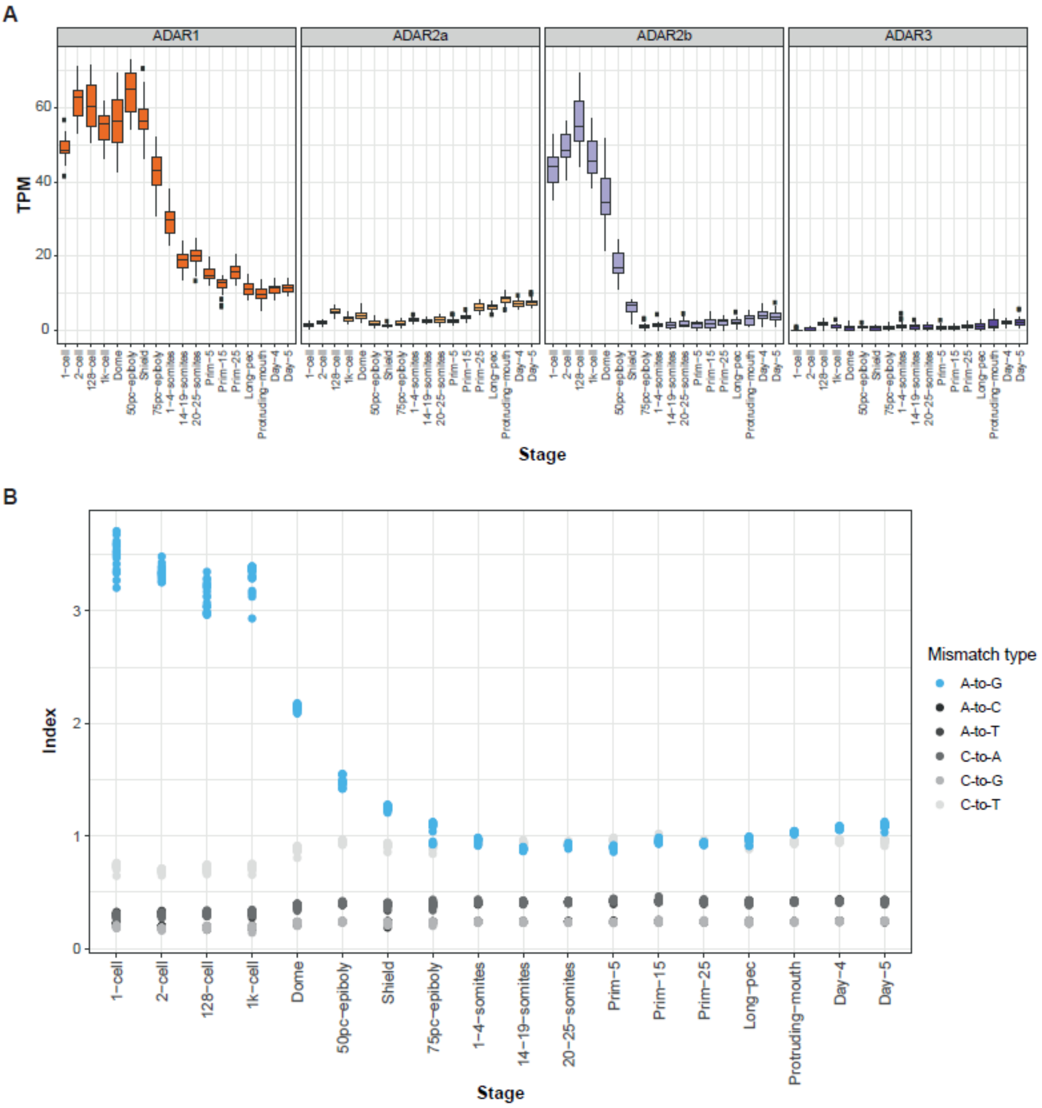

Supp. Figure 4: RNA editing during zebrafish development. Analyses of publicly available dataset (PRJEB12982) from 18 time points along the embryonic development in zebrafish **(A)** ADAR expression levels of ADAR1, ADAR2a, ADAR2b and ADAR3 suggest that ADAR1 and ADAR2b are overexpressed in the initial steps of embryonic development. **(B)** Distribution of repeats editing index values over developmental stages. High levels of editing are detected in early developmental stages, with a similar pattern of ADAR1 and ADAR2b expression.

Supplementary figure 5

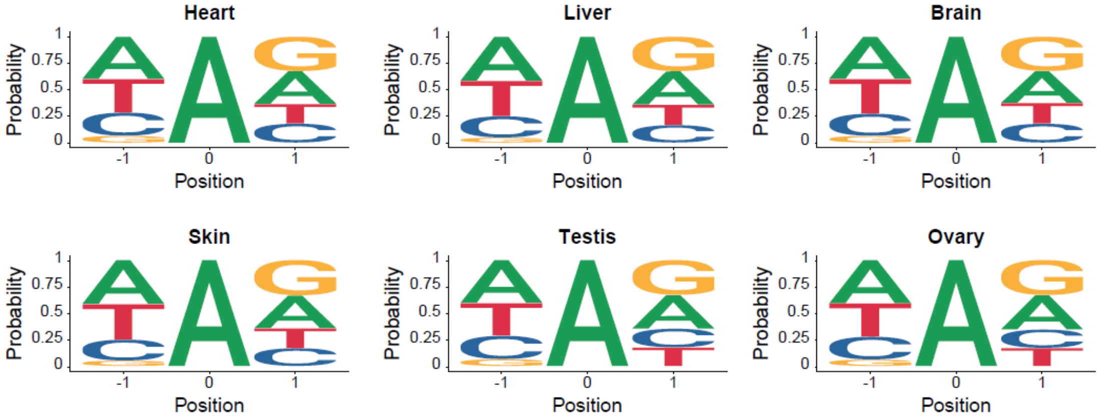

Supp. Figure 5: ADAR motif. All editing sites exhibited the known ADAR motif across tissues, with a strong depletion of guanosine (G) immediately upstream of the edited site, and some enrichment of G immediately downstream

Supplementary figure 6

A

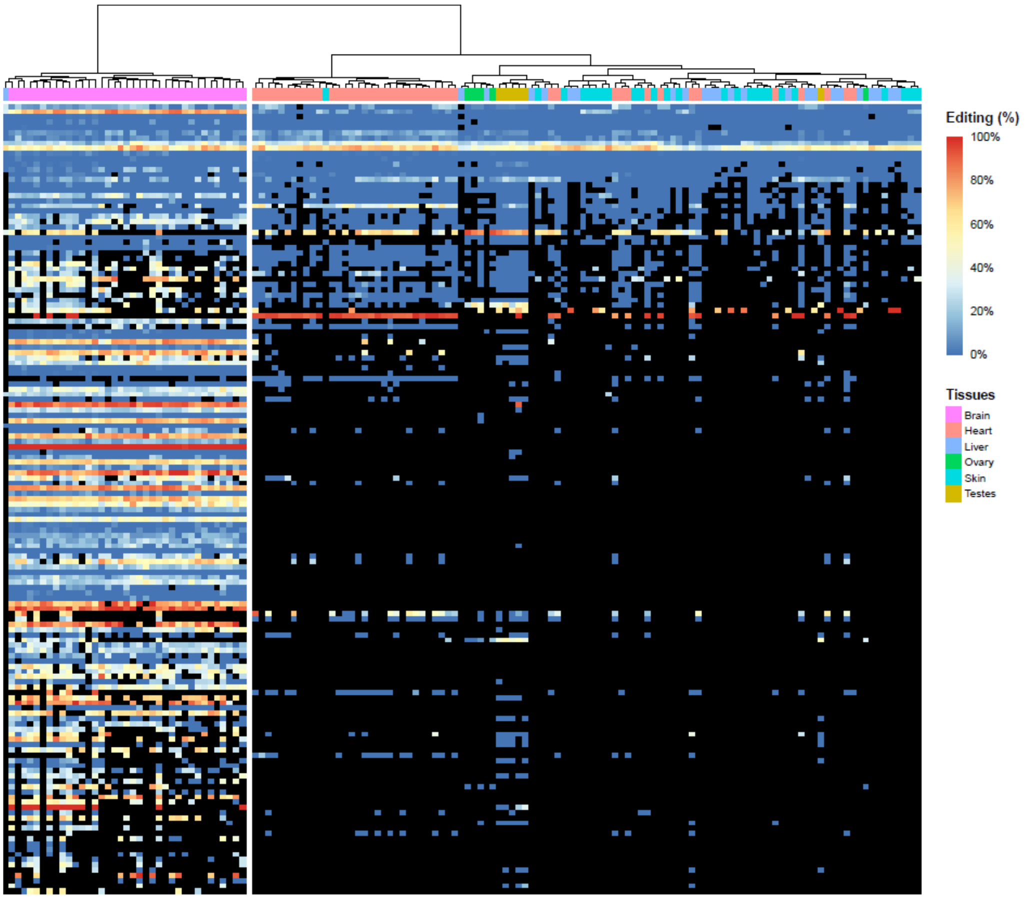

B

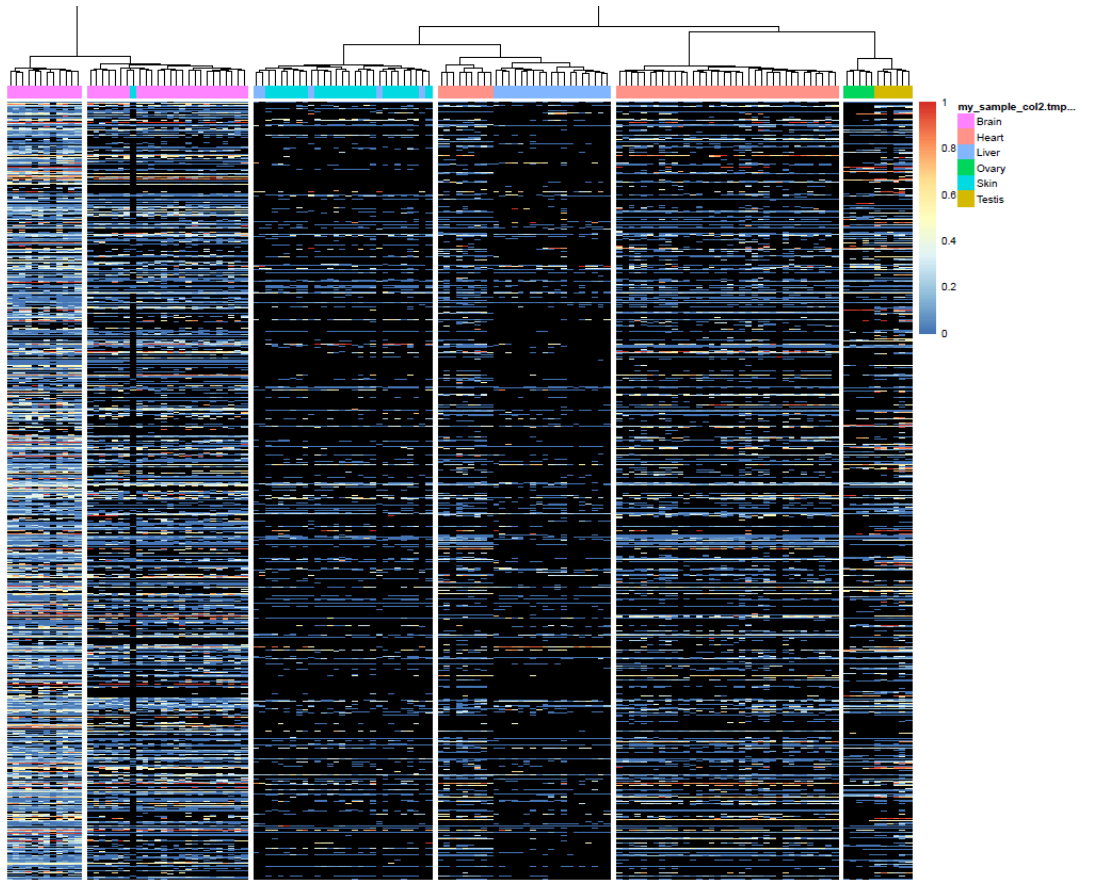

Supp. Figure 6: Heat map of RNA-editing frequency of putative A-to-I editing sites. Only sites covered with more than 10 reads are shown. The color of each rectangle represents the editing level (white denotes 0% editing; blue denotes 100% editing). Black rectangles denote editing sites supported by less than 10 reads or those that had no coverage. **(A)** Clustering analysis of the 149 detected coding editing events across tissues, revealed a clear separation between brain and non-brain tissues **(B)** Clustering analysis of 757,717 putative editing sites revealed an almost perfect separation between tissues.

Supplementary figure 7

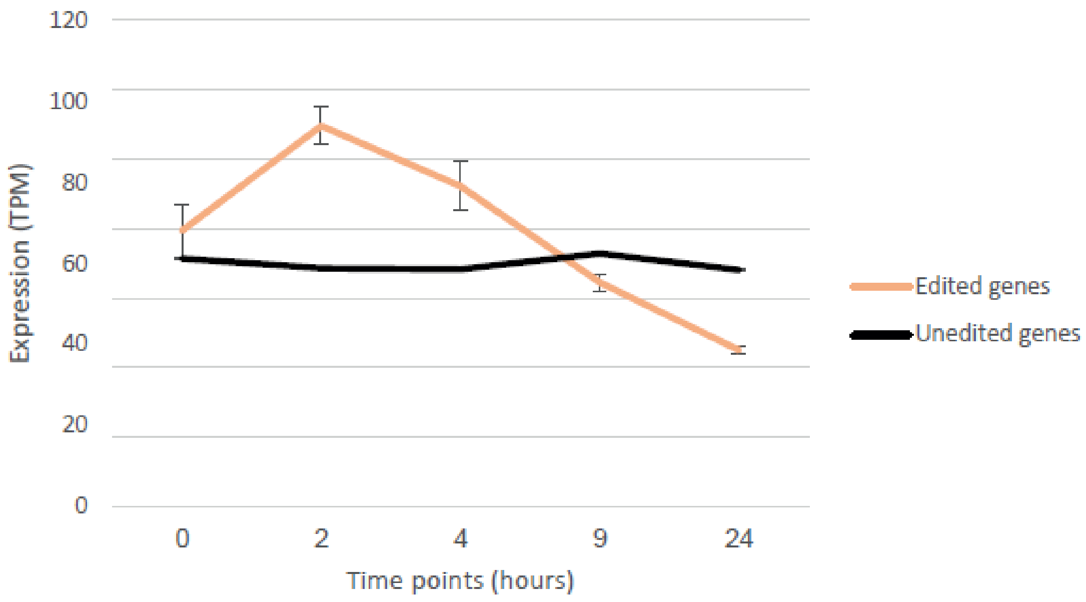

Supp. Figure 7: Transcript expression levels during zebrafish development. Comparison of transcripts' mean expression levels of genes that were edited at 0 hours (253 genes) versus the same genes at 2, 4, 9 and 24 hours. Expression levels of edited genes at 0h decreases during developmental stages, comparing to unedited genes. Thus, it is possible that RNA editing of those genes impact the mRNA stability in early embryonic stages, yet, further analyses are needed.
